# Genetic and biochemical differences in populations bred for extremes in maize grain methionine concentration

**DOI:** 10.1186/1471-2229-14-49

**Published:** 2014-02-19

**Authors:** Mark A Newell, Karla E Vogel, Marie Adams, Nevzat Aydin, Anastasia L Bodnar, Muhammad Ali, Adrienne N Moran Lauter, M Paul Scott

**Affiliations:** 1 The Samuel Roberts Noble Foundation, Ardmore, Oklahoma 73401, USA; 2 Iowa State University, Interdepartmental Genetics graduate program, Ames, IA 50011, USA; 3 Monsanto Company, St Louis, MO 63137, USA; 4 Bioengineering Department, Karamanoglu Mehmetbey University, Faculty of Engineering, Karaman 70100, Turkey; 5 North West Frontier Province Agricultural University, Peshawar, Pakistan; 6 USDA-ARS, Corn Insects and Crop Genetics Research Unit, Ames, IA 50011, USA

**Keywords:** Methionine, Breeding, Association mapping, Sulfur assimilation, Storage proteins

## Abstract

**Background:**

Methionine is an important nutrient in animal feed and several approaches have been developed to increase methionine concentration in maize (*Zea mays* L.) grain. One approach is through traditional breeding using recurrent selection. Using divergent selection, genetically related populations with extreme differences in grain methionine content were produced. In order to better understand the molecular mechanisms controlling grain methionine content, we examined seed proteins, transcript levels of candidate genes, and genotypes of these populations.

**Results:**

Two populations were selected for high or low methionine concentration for eight generations and 40 and 56% differences between the high and low populations in grain methionine concentration were observed. Mean values between the high and low methionine populations differed by greater than 1.5 standard deviations in some cycles of selection. Other amino acids and total protein concentration exhibited much smaller changes. In an effort to understand the molecular mechanisms that contribute to these differences, we compared transcript levels of candidate genes encoding high methionine seed storage proteins involved in sulfur assimilation or methionine biosynthesis. In combination, we also explored the genetic mechanisms at the SNP level through implementation of an association analysis. Significant differences in methionine-rich seed storage protein genes were observed in comparisons of high and low methionine populations, while transcripts of seed storage proteins lacking high levels of methionine were unchanged. Seed storage protein levels were consistent with transcript levels. Two genes involved in sulfur assimilation, *Cys2* and *CgS1* showed substantial differences in allele frequencies when two selected populations were compared to the starting populations. Major genes identified across cycles of selection by a high-stringency association analysis included *dzs18*, *wx*, *dzs10*, and *zp27*.

**Conclusions:**

We hypothesize that transcriptional changes alter sink strength by altering the levels of methionine-rich seed storage proteins. To meet the altered need for sulfur, a cysteine-rich seed storage protein is altered while sulfur assimilation and methionine biosynthesis throughput is changed by selection for certain alleles of *Cys2* and *CgS1*.

## Background

Methionine is an essential amino acid that is frequently supplemented in animal diets, especially those of poultry. Genetic approaches to increase the level of methionine in maize (*Zea mays* L.) grain would reduce the level of supplementation required, decreasing the cost of animal diets. These approaches would be facilitated by an understanding of molecular mechanisms controlling methionine deposition in the grain.

Methionine and cysteine are the major sulfur-containing amino acids; therefore levels of grain methionine may be related to sulfur uptake and/or assimilation. Sulfur uptake and assimilation are tightly regulated and coordinated processes allowing plants to respond to variations in sulfur levels in the environment [1]. Sulfate assimilation in plants has been reviewed [2]. Briefly, sulfate from the environment is activated to 5′-adenlylysulfate by the enzyme adenosine - 5′-triphosphate sufurylase. This activated sulfur is then reduced to sulfide that is fixed in the form of cysteine by o-acetyl-L-serine (thiol)lyase, also known as cysteine synthase, which is encoded by the *Cys2* gene in maize. The committed step in the production of methionine is a cysteine-dependent reaction catalyzed by cystathione gamma-synthase, *CgS1*. The *Cys2* gene may have been a target of selection in the course of improvement of modern maize inbreds based on the observation that it has low diversity among maize and teosinte varieties [3]. However, a study focusing on genes in amino acid biosynthesis [4] concluded that genes with reduced diversity tend to be clustered in a few pathways and most amino acid biosynthesis genes have sufficient diversity for continued improvement through breeding.

A second factor that influences grain methionine concentration is the presence of abundant, methionine-rich proteins in the grain. Prolamin seed storage proteins of maize are called zeins and influence the properties of grain due to their abundance. For example, increased levels of the transcript of the 10 kDa delta zein, encoded by *dzs10*, have been shown to be responsible for the high methionine concentration in the inbred line BSSS-53 [5]. A second methionine-rich delta zein gene, designated *dzs18*, has also been identified [6] that exhibits variation in expression among maize inbreds and teosinte. Thus, grain methionine concentration is potentially regulated by mechanisms related to source strength including uptake and assimilation and by mechanisms related to sink strength including levels of certain seed storage proteins.

An understanding of factors controlling grain methionine concentration has facilitated several genetic approaches to increase levels of methionine in maize grain. For example, the allele responsible for a high *dzs10* transcript level in BSSS-53 was identified by screening seedlings for resistance to high levels of lysine and threonine in germination medium [7]. This allele has served as the basis for development of other high methionine inbred lines [8]. In addition, it has been shown that this allele functions by increasing the stability of the *dzs10* transcript via a post transcriptional mechanism [9] and is controlled by a locus called *dzr1*, which is not genetically linked to *dzs10*[9-11]. Characterization of this mutation has led to the development of a transgenic approach that was used to increase methionine concentration [12].

Recurrent selection has been shown to be an effective method for increasing methionine concentration of grain [13]. Divergent selection for high and low grain methionine concentration was carried out in two starting populations, BS11 and BS31. These two populations are broad-based synthetics that have been selected for agronomic traits. Three cycles of selection for high and low grain methionine level resulted in populations with a change in methionine concentration of about 0.014 g/100 g per cycle of selection. After three generations of selection, the ratio of methionine concentration in high to low methionine populations was about 1.3. Populations divergent for traits of interest are also conducive to analyses that capitalize on an increase in detection power due in large part to differences in phenotype [14].

Our objective is to gain a better understanding of the molecular basis of response to selection for grain methionine concentration. Our approach was to carry out recurrent selection for high and low grain methionine concentration through eight cycles of selection and characterize the resulting populations genetically and biochemically. We show that the advanced cycles of selection exhibit extreme differences in grain methionine concentration between the high and low methionine populations. In order to gain some information about the genetic control of methionine concentration, we conducted an association analysis to identify SNPs significantly associated with methionine concentration. In addition, we carried out molecular and biochemical characterization of the selected populations, including characterization of transcript levels of genes involved in sulfur assimilation as well as transcript and protein levels of seed storage proteins. Our observations provide insights into physiological and genetic mechanisms controlling grain methionine concentration in maize.

## Methods

### Populations used in the study

Recurrent selection was carried out starting with two unrelated maize populations. One population was derived from BS11 which was originally called the Pioneer two-ear composite and was developed by crossing southern prolific material and corn belt lines [15]. The second population was derived from BS31, which was originally called FS8B and was developed by mass selection for earliness in a synthetic containing tropical germplasm [16]. The recurrent selection experiment used to develop the populations for this study is outlined in [13]. The selection process was carried out for 8 generations.

### Genotyping

Remnant kernels were available from most of the selected ears as well as kernels from the original BS11 and BS31 random-mated populations and these kernels were used for genotyping as follows. From each population, ten individuals were genotyped from each of the five selected ears that constituted that population. Thus, 50 individuals were genotyped for each population (illustrated in Additional file 1). In total, 505 individuals from BS11-derived populations were included in this study as follows. Fifty individuals from each high or low methionine population from cycles two through five were analyzed. In addition, 105 individuals from BS11C0 were included in the analysis. Similarly, fifty individuals from each selected population derived from BS31 were analyzed. These populations included C1, C3, C4, C5, and C6. Remnant seed was not available for BS31LMC2 BS31LMC4 and BS31HMC2. An additional 125 individuals from BS31 were analyzed bringing the total sample count to 575.

For each population, fifteen kernels were grown out as follows. Germination paper was dampened with water and the fifteen kernels were placed at the top of the paper. The paper was then folded to cover the kernels. After loosely rolling the paper into a tube and labeling with the pedigree information, the tube was placed upright in a few inches of water to allow the roots to grow downward. After 12 days at ambient temperature leaf tissue was collected by tearing a piece of leaf (equivalent to size of approximately 1/2″ × 1/2″) from each plant and placing it in an individual well of a 96-well plate. This process was repeated with 10 plants from each selected population. Leaf tissue was collected from all seedlings that emerged in the starting populations.

Samples were then processed through Monsanto’s high throughput genotyping facility in Ankeny, Iowa. Fully automated genotyping systems were used to process samples from DNA extraction through allele calling. DNA samples were genotyped for a set of biallelic single-nucleotide polymorphism (SNP) markers using the Taqman assay described by Livak [17]. For quality control purposes, eight standards of known genotypes were assayed per 184 samples genotyped.

The molecular markers used were chosen from the Monsanto consensus map based on their polymorphic information content value. This consensus linkage map utilizes an intermated B73 × Mo17 population to map the locations of individual SNPs on each of maize’s ten chromosomes [18]. Two groups of markers were used in this study. Markers in the first group were chosen to obtain maximal genome coverage. As shown in Additional file 2, 121 markers were chosen throughout the genome with an average distance between markers of 13.8 cM. Assuming individual marker coverage of 10 cM, this marker set represents approximately 82% genome coverage. Markers in the second group were chosen to obtain thorough coverage in six regions of interest that contain candidate genes involved in sulfur assimilation, methionine biosynthesis, or methionine storage. The waxy gene region was also included in this study as a reference region. Each of these regions was densely covered with SNP markers with an average distance of 2.4 cM. Genome-wide markers are labeled Mx, where x is a sequential number. Gene specific markers are notated with the gene name, dash, and sequential number. A total of 169 SNP molecular markers were applied to each sample in this study (Additional file 2).

### Compositional analysis of grain

Methionine in mature maize grain was quantified using a high-throughput microbiological method described by Scott et al. [19]. In the course of selection in cycles 6, 7 and 8 in 2007, 2008 and 2010, respectively, approximately 40 kernels from each of the 50 ears in each population were ground using a Stein grinder in order to create a sample representative of each ear. Ten mg of the ground grain was then deposited into a randomly assigned well of a 96-well plate. The protein in this grain was hydrolyzed by incubation with pepsin at pH2, and the resulting hydrolysate was added to a plate that was inoculated with the P4X *E. coli* strain, which is auxotrophic for methionine [20]. This plate was then shaken at 37°C for 16 hours. Following incubation, the optical density at 595 nm was measured with a Tecan plate reader. These data are presented in Table 1. In addition, Analysis of the cycle 8 populations was carried out using the AOAC standard method for methionine analysis by creating an equal mass bulk of ground grain from 10 ears for each population. Analysis was carried out by the University of Missouri Experiment Station Chemistry laboratory. Total protein was evaluated by combustion analysis at the Iowa State University Plant and Soil Analysis Laboratory using a Leco TruSpec CN (St. Joseph, MI). Sulfur analysis was carried out by microwave digestion of the ground bulks [21] followed by ICP-Atomic Emission Spectrometry using a Spectro Ciros CCD ICP-AES (Spectro Analytical Instruments, Mahwah, NJ). These data are presented in Table 2.

**Table 1:** Grain methionine concentration in high and low methionine populations

**Starting population**	**Cycle**	**Year**	**HM mean (S.D.)**^**1**^	**LM Mean (S.D.)**	**Difference (S.D.)**
BS11	6	2007	0.14	−0.43	0.57
	7	2008	0.73	−0.79	1.52
	8	2010	0.69	−0.86	1.39
BS31	6	2007	0.72	−0.81	1.53
	7	2008	0.92	−0.74	1.66
	8	2010	0.76	−0.63	1.11

^1^Mean of the 50 ears in the high and low populations determined by the microbial method and expressed in Standard Deviations from the mean of the union of the high and low populations.

**Table 2:** Mean grain amino acid, total nitrogen, and total sulfur concentration of 10 pooled C8 individuals

	**BS11 HM**	**BS11LM**	**Ratio HM/LM**	**BS31HM**	**BS31LM**	**Ratio HM/LM**
Taurine^1^	0.06	0.06	1.00	0.06	0.06	1.00
Hydroxyproline	0.02	0.02	1.00	0.01	0.02	0.50
Aspartic acid	0.73	0.69	1.06	0.66	0.73	0.90
Threonine	0.39	0.35	1.11	0.36	0.39	0.92
Serine	0.49	0.44	1.11	0.47	0.49	0.96
Glutamic acid	2.12	1.84	1.15	2.00	2.12	0.94
Proline	0.93	0.83	1.12	0.90	0.93	0.97
Glycine	0.42	0.40	1.05	0.39	0.42	0.93
Alanine	0.86	0.76	1.13	0.81	0.86	0.94
Cysteine	0.22	0.18	1.22	0.22	0.22	1.00
Valine	0.55	0.51	1.08	0.49	0.55	0.89
Methionine	0.25	0.16	1.56	0.28	0.25	1.12
Isoleucine	0.41	0.38	1.08	0.37	0.41	0.90
Leucine	1.46	1.26	1.16	1.36	1.46	0.93
Tyrosine	0.35	0.30	1.17	0.33	0.35	0.94
Phenylalanine	0.58	0.51	1.14	0.52	0.58	0.90
Hydroxylysine	0.01	0.02	0.50	0.02	0.01	2.00
Ornithine	0.01	0.01	1.00	0.01	0.01	1.00
Lysine	0.34	0.36	0.94	0.31	0.34	0.91
Sum of AAs	11.06	9.94	1.11	10.38	10.85	0.96
Nitrogen (%)^2^	1.58	1.24	1.27	1.40	1.43	0.98
Total S (ppm)^3^	874	495	1.76	952	787	1.21

^1^Amino acid concentrations are in mass percentage of the amino acid in the tissue analyzed.

^2^% Nitrogen was determined by combustion analysis.

^3^Total sulfur content was determined by ICP.

### Association analysis for methionine concentration

Principal components analysis (PCA) was used to explore the divergence across populations used in this study. In order to compute PCA, a common SNP marker set across the BS11- and BS31-derived populations was identified and merged. The final data set used for PCA consisted of 102 SNPS and 1,055 lines. By using this approach for PCA, the derived populations and cycles of selection could be easily compared.

Association analysis [22] was implemented to identify SNPs associated with increased methionine concentration. The model used was *Y = mean + Ms + Pv + Zu + e* where *Y* is a vector of classification for the HM or LM populations, *s* is a vector of SNP effects, and *u* is a vector of polygenic random effects. The variances of the random polygenic effects is assumed to be Var(u) = 2KVg where K is a square matrix of relative kinship coefficients and Vg is the genetic variance. A classification was used as the response variable, *Y*, because methionine level was not measured for each genotyped line individually. The Benjamini and Hochberg false discover rate (FDR) of 0.01 was applied for multiple testing of significant SNPs. Such a high stringency was used due the fact that the association analysis was conducted by means of a candidate gene approach. The model for association analysis was implemented in the rrBLUP [23] package within the R statistical software [24]. For the BS11-derived population, C2, C3, C4, and C5 were analyzed for significant SNPs on an initial data set of 158 SNPs. For the BS31-derived population, C2, C3, C5, and C6 were analyzed for significant SNPs on an initial data set of 102 SNPs. C4 was not analyzed for the BS31-derived population because of missing data for the LM population. Thus, eight analyses were conducted across starting populations and cycles. For significant SNPs, the level of linkage disequilibrium (LD), as the correlation squared, was calculated to gain an understanding of their relationships.

### Determination of candidate gene allele frequencies by sequencing

Genomic DNA from approximately 50 individual plants from the cycle 6 populations was extracted from individual maize seedlings using a protocol scaled for 96-well plates (N. Lauter, personal communication). This DNA was used as template for a PCR using primers specific for the candidate genes of interest. The resulting PCR products were sequenced from both ends at the Iowa State University DNA Facility using the Applied Biosystems 3730×l DNA Analyzer (Applied Biosystems, Foster City, CA) and the resulting sequences were aligned using the BioEdit Sequence Alignment Editor [25]. The ClustalW alignment application was used with optimal settings to align the resulting sequences and the haplotype(s) in each plant present was determined by visual inspection of the alignments. Heterozygotes were identified by visual inspection of the sequencing data. Deviation from Hardy-Weinberg equilibrium was determined using a χ^2^ test.

### Transcript quantitation

In 2005, 2008 and 2010 corresponding to cycles 4, 6 and 8 respectively, immature endosperm tissue was collected at 18 days after pollination from between 3 and 10 plants from each population (Table 3). Polyadenylated mRNA was isolated from approximately 100 mg of each endosperm tissue collected using the Promega PolyATtract 1000 kit (Promega Corp., Madison, WI) without modification. Isolated RNA was quantified using a NanoDrop ND-1000 Spectrophotometer and stored at −80°C until amplification reactions could be performed.

**Table 3:** Ratios (HM/LM) in transcript levels of candidate genes in developing endosperm

**Year**	**2005**	**2008**	**2008**	**2010**	**2010**
**Population**	**BS11C4**	**BS11C6**	**BS31C6**	**BS11C8**	**BS31C8**
Genes					
ATPSase	n.d.^1^	n.d.	0.45	1.43	2.70*
*CgS1*	n.d.	0.63	1.76	0.62	1.80*
*Cys2*	1.01	0.48	2.67	0.72	1.21
27 kDa γ-zein	1.07	0.42	4.32	0.17**	0.61
*dzs10*	2.36	3.89*^2^	4.56	3.8*	4.65**
*dzs18*	2.69	19.30*	9.71*	6.50**	9.53**
19kD α B1	2.11	0.49	2.43	1.91	0.77
15 kDa zein	n.d.	0.57	7.26*	0.78	2.69*
16 kDa zein	7.64	0.78	3.19*	1.97*	1.49
*Fl2*	1.69	1.44	12.55	1.57	1.06
Reps H/L	7/7	7/3	7/6	10/10	10/10

^1^n.d. Not determined.

^2^* p < .05, ** P < 0.1.

Real-time quantitative polymerase chain reaction experiments were performed with 1 ng of endosperm mRNA as an initial template. Stratagene Brilliant II QRT-PCR kit was used to make cDNA and carry out PCR in 25ul volumes as described in the manual with 200 nM forward and reverse primers.

Cycling protocols were performed in a Stratagene MX3000P (Stratagene, La Jolla, CA) thermocycler and consisted of a first strand synthesis of 45°C for 45 min, followed by 95°C for 10 min, followed by 40 cycles of 95°C for 30 sec, 59°C for 1 min, 72°C for 45 sec, followed by a dissociation curve. The delta-delta Ct value was calculated using either actin or the 18S ribosomal RNA as the internal control [17].

### Analysis of alcohol-soluble protein concentration

Alcohol-soluble proteins were analyzed by high pressure liquid chromatography as described previously [26]. Grain from seven randomly selected individuals from BS11LMC6 and six randomly selected individuals of BS11HMC6 was ground for analysis. Alcohol-soluble proteins were extracted from 25 mg of flour using 250 μL extraction buffer consisting of 70% EtOH, 61 mM NaOAc, and 5% β-mercaptoethanol. The mixture was vortexed briefly, shaken for 1 h at room temperature, and then centrifuged for 10 min at 13,000 rpm. Each extract was diluted 1:4 in extraction buffer and an aliquot of 25 μL was injected into a C18 protein and peptide column heated to 55°C in a Waters 2695 Separation Module, and absorbance at 200 nm was measured with a Waters 2487 Dual Absorbance Detector. Separation of distinct proteins based on hydrophobicity was achieved with a gradient of ultrapure water and acetonitrile, both containing 0.1% trifluoroacetic acid. The complex gradient ranged from 20 to 60% acetonitrile for a total of 96 min excluding equilibration steps before and after elution. Gradient slopes were optimized for separation of peaks. A flow rate of 1 mL/min to 0.5 mL/min was used. Chromatographs for all individuals were averaged by population to produce one chromatogram for BS11HMC6 and one for BS11LMC6. These two chromatograms were subtracted to examine differences in the two populations. The delta zein peaks were assigned by analysis of characterized mutants [27] and other peaks were assigned by comparison to previously published information [28].

## Results

In order to generate populations with highly contrasting values for methionine, we continued the previously described recurrent selection program [13] using the same procedure for a total of eight cycles of recurrent selection. In this program, divergent mass selection was implemented for high and low grain methionine concentration in two independent starting populations. This led to four selection tracks, with populations in each track designated according to the following convention: BS11 or BS31, depending on the starting population; HM or LM, depending on whether selection was for high or low methionine concentration; C# indicating the cycle of selection. Thus, BS11HMC8 is the eighth cycle of selection for high methionine for the BS11-derived population.

### Grain composition in selected populations

Grain methionine concentration was initially evaluated after three cycles of recurrent divergent selection where large differences between HM and LM were observed [13]. In cycles 6, 7 and 8, 50 individuals from both high and low populations were evaluated in order to make selections. For this purpose, precision in determining sample ranks is more important than accuracy, so we used a high-throughput microbial assay and report the results on the basis of standard deviations from the mean of the HM and LM populations (Table 1). In all cases the mean of the HM population was higher than the mean of the LM population by at least 0.57 standard deviations. The largest difference observed was 1.66 standard deviations for the BS31-derived population at cycle 7. It should be noted that the data from each cycle were produced in different environments, so comparisons should only be made between the HM and LM populations of a given cycle. While we cannot determine if methionine concentration is diverging with continued selection in the HM and LM populations, these data show there is a significant difference between methionine concentration for the HM and LM populations in each year.

To verify the observations made with the microbial assay and to obtain absolute values of methionine content, we evaluated the most advanced populations using the standard AOAC method of analysis (Table 2). The amino acid with the highest ratio of HM/LM was in each population methionine, with ratios of 1.54 and 1.12 for the BS11- and BS31- derived populations, respectively. This is consistent with the observations made using the microbial method. The amino acid with the next highest ratio was cysteine in the BS11-derived population with a value of 1.22. These data illustrate the remarkable specificity of selection for grain methionine concentration, whereby the largest changes were observed for methionine, and observed differences for other amino acids were much smaller.

One way to increase methionine concentration would be to increase the total protein concentration of the seed. Summation of the amino acid concentrations in Table 2 can be used to approximate total protein concentration. In addition, we measured nitrogen concentration on the same material (Table 2). The results of these two measurements are in agreement, both showing a higher total protein concentration in the BS31HM population than in BS31LM and a lower total protein concentration in the BS11HM than in the BS11LM population. These changes are not sufficiently large (in the case of the BS11-derived populations) or in the right direction (in the case of the BS31-derived populations) to explain the observed differences in methionine concentration. Thus we conclude that the change in methionine concentration is not due to an increase in total protein concentration. This is in contrast to the total sulfur content in the grain (Table 2), which was clearly higher in both high methionine populations examined compared to their low methionine counterparts. The magnitudes of the HM/LM ratios are similar to those for methionine, with a higher ratio occurring in the BS11 populations and the lower ratio in the BS31 populations.

### Association analysis and linkage disequilibrium

**Figure 1:**
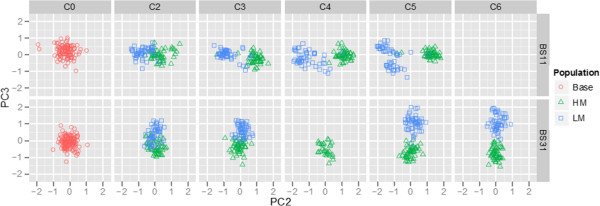
**Principal component two (PC2) versus PC3 for the BS11- and BS31-derived populations.** The divergence of HM (green) and LM (blue) from cycle two (C2) to C6 from the base population (red) increases with each cycle of selection. We do not have data for BS31C4LM, BS11C6HM and BS11C6LM.

In order to better understand the genetic control of methionine content, we set out to identify genetic loci associated with this trait. We first set out to obtain an overview of the genetic architecture of the derived populations across cycles of selection. We did this by genotyping each population using single nucleotide polymorphisms (SNPs) from C0 to C6 depending on the derived population. Principal component analysis (PCA) was used to explore the divergence of the HM and LM populations for the BS11- and BS31-derived populations using a set of SNPs common to both populations. The first three principal components accounted for 22.00, 5.25, and 3.72% of the variation in the data, respectively. Interestingly, the BS11- and BS31-derived populations were separated by PC1, the HM and LM populations within the BS11-derived population were separated by PC2, and the HM and LM populations within the BS31-derived population were separated by PC3 (Figure 1). This analysis suggests that divergence in methionine content was accompanied by genetic divergence as well. The observation that divergence in the BS11-derived populations aligns with PC2 while divergence of BS31-derived populations aligns with PC3 suggests that the genetic divergence of the two populations is driven by different sets of loci. This could indicate that a change in methionine concentration is affected by different molecular mechanisms in the two derived populations.

**Figure 2:**
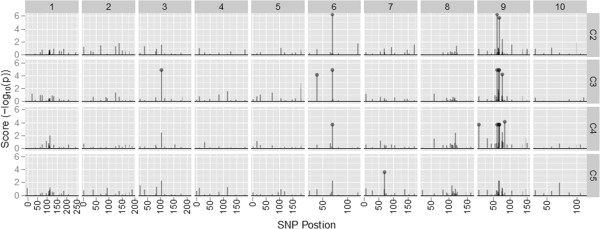
**Ordered SNPs versus the association score for each SNP. **Data is from the BS11-derived populations. Chromosome number is shown at the top and cycle of selection is shown on the right axis. Points represent significant SNPs at FDR < 0.01.

**Figure 3:**
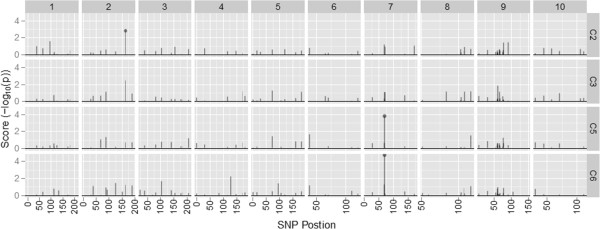
**Ordered SNPs versus the association score for each SNP. **Data is from the BS31-derived populations. Chromosome number is shown at the top and cycle of selection is shown on the right axis. Points represent significant SNPs at FDR < 0.01.

In order to gain a better understanding of the molecular changes that accompanied alteration in methionine concentration, we next determined which of the SNPs were significantly associated with the classification of populations according to their direction of selection, i.e. HM vs LM. Some SNPs may be associated with this classification as a result of drift in each cycle, but it would be unlikely that the same SNPs would be subject to drift in different cycles and in the two different populations. Eighteen SNPs were identified as significant in the BS11-derived population, whereas only three were identified for the BS31-derived population (Table 4). These SNPs were identified with a high degree of confidence, considering that a false discovery rate (FDR) of 0.01 resulted in the identification of only 11 different SNPs in BS11 and two different SNPs in BS31. There was commonality of SNPs significantly associated with methionine classification across the cycles of selection. For the BS11-derived population, dzs18.3, wxy.4, and the *dzs10* SNPs were consistent for more than one cycle of selection. Likewise, for BS31, the zp27.4 SNP was consistent for more than one cycle of selection. The SNP zp27.4 was significantly associated with methionine classification in both the BS11- and BS31-derived populations, an observation seemingly at odds with the conclusions of the principal component analysis. One locus is apparently not sufficient to cause divergence in the two populations to be explained by the same PC.Several of the SNPs that were significantly associated with HM and LM were localized on chromosome nine for the BS11-derived population (Figure 2). Interestingly, the significant SNPs on chromosome nine were only significant in C2, C3, and C4. The remaining significant SNPs for the BS11-derived population were localized on chromosomes three, six, and seven. In contrast, significant SNPs were localized to only chromosomes two and seven for the BS31-derived population (Figure 3).

**Table 4:** Significant SNPs associated direction of selection (HM or LM)

**Population**	**SNP**	**Chromosome**	**Position**	**Cycle**	**Score**^**1**^	**q-value**^**2**^
BS11	M38	3	104.0	C3	4.89	0.000092
	M71	6	39.9	C3	4.15	0.000461
	dzs18.3	6	70.0	C2	6.18	0.000005
				C3	4.89	0.000092
				C4	3.69	0.001052
	zp27.4	7	70.1	C5	3.55	0.002008
	M100	9	8.3	C4	3.69	0.001052
	wxy.4	9	62.1	C2	6.18	0.000005
				C3	4.89	0.000092
				C4	3.69	0.001052
	dzs10.6	9	67.0	C3	4.89	0.000092
				C4	3.69	0.001052
	dzs10.8	9	68.5	C2	5.63	0.000017
				C3	4.89	0.000092
				C4	3.69	0.001052
	dzs10.9	9	68.5	C4	3.69	0.001052
	cgs1.4	9	77.5	C3	4.21	0.000426
	M104	9	84.8	C4	4.11	0.000494
BS31	M25	2	165.2	C2	2.76	0.006289
	zp27.4	7	70.1	C5	3.78	0.000561
				C6	4.68	0.000112

^1^Score is calculated as the –log_10_(*p*), higher values indicate more evidence of a SNP associated with methionine level.

^2^*q*-value using the Benjamini and Hochberg method for multiple testing. A value of FDR < 0.01 was considered significant.

**Figure 4:**
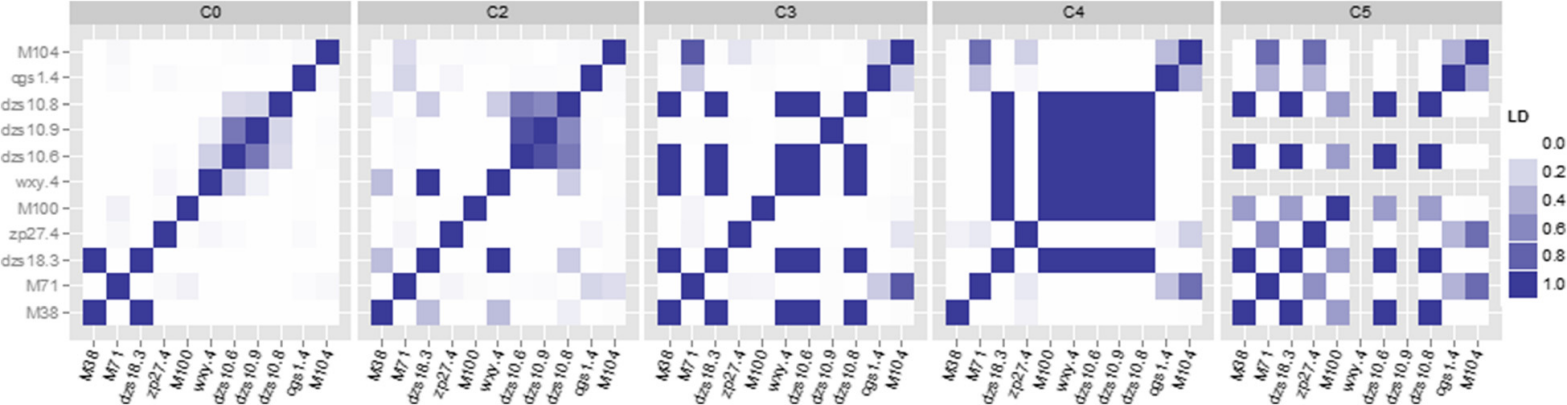
**Linkage Disequilibrium (LD), measured as r**^**2**^**, among significant SNPs identified in the BS11-derived population.** Alleles for wxy.4 and dzs10.9 are fixed in C5, therefore pair-wise LD could not be calculated for these SNPs.

One outcome of selection is the development of linkage disequilibrium between co-inherited alleles at different loci. We therefore examined the LD in each cycle of selection for each of the loci identified as being significantly associated with HM and LM in the BS11-derived population. A group of SNPs in LD, termed an LD block, can indicate the co-inheritance, or co-selection, of alleles at those particular loci. This was explored for the BS11-derived population, for the 11 different SNPs associated with methionine concentration, across cycles of selection, multiple LD blocks between linked and unlinked SNPs were observed (Figure 4). As expected, an increase of LD between the significant SNPs was observed through cycles of selection. In C0 an LD block was observed within the *dzs10* region including the *dzs10* SNPs and wxy.4. Additionally in C0, high LD was observed for between dzs18.3 and chromosome six and M38 on chromosome three. For C2 there was high LD observed between wxy.4 on chromosome nine and dzs18.3 on chromosome six in addition to the high LD among the *dzs10* SNPs. In C3 and C4, high LD was observed between the *dzs10* region including wxy.4 and dzs18.3. Finally, in C5 high levels of LD are observed among many of the linked and unlinked SNPs significantly associated with methionine concentration. In general, there is a clear trend toward increased LD with cycle of selection among the genes significantly associated with HM vs. LM, further supporting the hypothesis that the divergence observed in the principal component analysis is a product of selection.

### Transcript levels of genes involved in methionine metabolism in selected populations

In an effort to understand the physiological differences between populations selected for high and low grain methionine concentration, we examined transcript levels in mid-maturation endosperms (15 to 20 days after pollination) of several genes known to be involved in sulfur assimilation, methionine synthesis, and deposition. *Cys2* and *ATPSase* are known to play key roles in sulfur assimilation. *CgS1* is involved in methionine biosynthesis while *dzs10*, *dzs18* and the 15 kDa beta zein genes encode prolamin seed storage proteins with about 20% methionine on a per residue basis. The *zp27* and *zp16* genes encode the 27 and 16 kDa gamma zeins, repetitively, which are prolamin seed storage proteins with a high concentration of cysteine while *Fl2* encodes a prolamin seed storage protein with 2% sulfur amino acids. Transcript levels were measured in populations representing three different cycles of selection where the tissue representing each cycle was produced in a different year. This design provides an overview of the variation in the experiment, but does not permit conclusions to be drawn about specific cycles of selection or the effect of environments. The results are summarized in Table 3. Transcripts involved in sulfur assimilation accumulated to higher levels in populations selected for high methionine than in populations selected for low methionine in the majority of the cases examined, however the results were not consistent across years or populations. By contrast, transcripts encoding high methionine seed storage proteins were up-regulated in populations selected for high methionine compared to populations selected for low methionine in a more consistent manner. The 27 kDa gamma zein was the only transcript that was down regulated significantly, and that was only in one of the four year/population combinations. The *Fl2* transcript was not significantly differentially expressed in any of the conditions examined. Since the *Fl2* protein does not have elevated levels of sulfur containing amino acids, this observation supports the hypothesis that the changes in seed storage protein transcripts are confined to transcripts of proteins with high concentration of sulfur amino acids.

### Seed storage protein concentrations in selected populations

**Figure 5:**
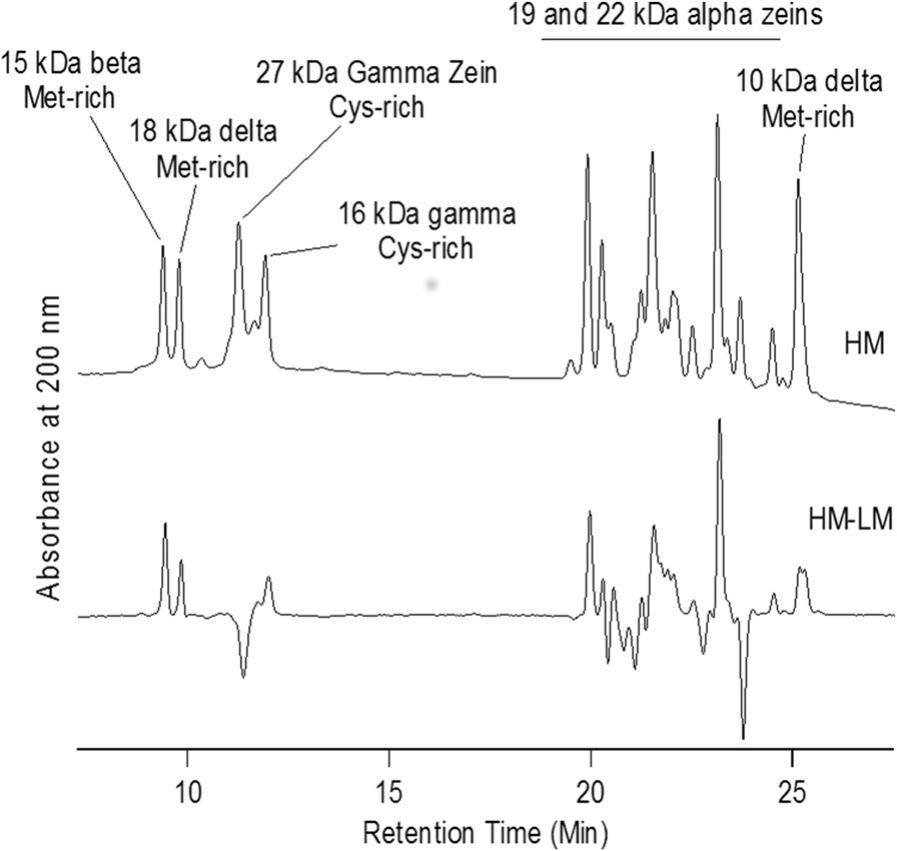
**HPLC chromatograms of alcohol soluble seed proteins.** Top trace: Average of 5 individuals from BS11HMC6 population. Bottom trace: Subtraction of Average of 6 individuals from the BS11HMC6 population minus the top trace. Peaks above the zero line are higher in the HM and below the zero line are higher in LM.

Since the seed storage protein transcripts showed differential expression in some cases, we examined levels of the corresponding proteins and other alcohol-soluble proteins (Figure 5). Several methionine-rich zeins accumulated to higher levels in the HM relative to the LM populations, including the 10 and 18 kDa delta zeins and the 15 kDa beta zein. The 27 kDa gamma zein is rich in cysteine and showed a marked reduction while the other cysteine-rich zeins showed a slight increase. The results for the 22 kDa alpha zein are difficult to interpret because of the complexity of this area of the chromatogram. It appears that some alpha zeins were increased and others were decreased.

### Allele frequencies of genes involved in methionine metabolism in selected populations

Since our starting populations were broad-based synthetics, it is likely that multiple alleles are present at some of our candidate gene loci. We examined allele frequencies at two loci, *Cys2* and *CgS1* in the BS11-derived population and after 6 cycles of selection for high and low methionine (Table 5). We identified five alleles at *Cys2* and two alleles at *CgS1*. One consequence of selection could be a departure from Hardy-Weinberg equilibrium at loci controlling the methionine concentration. *CgS1* did not have any alleles that differed significantly from the expected frequencies. In contrast, *Cys2* had alleles that differed from the expected frequencies in both the C0 and the HM populations.

**Table 5:** Allele frequencies of candidate genes

**Gene**	**Population**	**Allele**^**1**^	**Number of haplotypes**	**Number of homozygotes**	**Expected homozygotes**^**2**^	**P value**^**3**^	**Significance**^**4**^
*Cys2*	C0	1	0	0	0	N/A	
		2	6	2	0	>0.01	**
		3	44	18	13	0.21	n.s.
		4	18	5	2	0.07	n.s.
		5	4	1	0	>0.01	**
	LMC6	1	0	0	0	N/A	
		2	0	0	0	N/A	
		3	20	6	3	0.05	n.s.
		4	52	22	19	0.46	n.s.
		5	0	0	0	N/A	
	HMC6	1	10	5	1	>0.01	**
		2	27	4	4	0.95	n.s.
		3	55	18	16	0.63	n.s.
		4	1	0	0	0.94	n.s.
		5	1	0	0	0.94	n.s.
*CgS1*	C0	1	21	7	11	0.23	n.s.
		2	17	5	7	0.41	n.s.
	LMC6	1	45	16	11	0.17	n.s.
		2	35	11	7	0.12	n.s.
	HMC6	1	5	0	0	0.68	n.s.
		2	67	31	31	0.98	n.s.

^1^Alleles were arbitrarily assigned numbers based on SNPs in the section of the gene that was sequenced.

^2^Number of homozygotes that would be expected under Hardy-Weinberg equilibrium.

^3^Probability that the observed deviation from Hardy-Weinberg equilibrium is due to chance based on a chi-squared test with one degree of freedom.

^4^Significance of the test for Hardy-Weinberg equilibrium: n.s., not significant; *, probability of observed deviation being due to chance < 0.05 or **,< 0.01.

**Figure 6:**
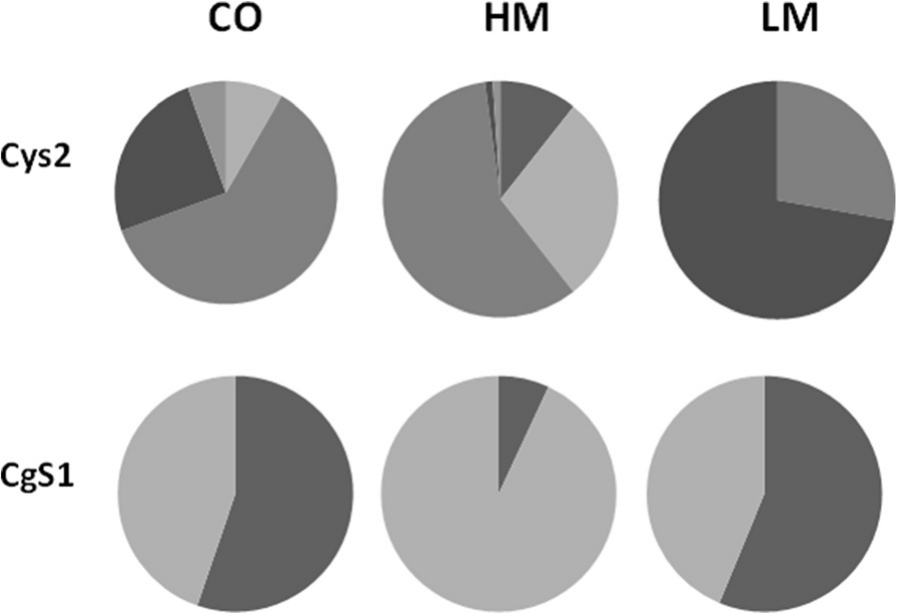
**Allele frequencies of candidate genes in the starting population (C0) and after 6 cycles of selection.***Cys2*: starting population, n = 72, high population, n = 94, low population, n = 72; *CgS1*: starting population, n = 19, high population, n = 36, low population, n = 40.

Recurrent selection would be expected to alter allele frequencies at loci that influence the trait under selection. Further, alleles that are enriched by selection for high methionine may be desirable alleles for increasing methionine concentration while alleles that are enriched by selection for low methionine may be undesirable. Changes in allele frequency were evident at both loci (Figure 6). At the *Cys2* locus, one of the alleles that started at 25% was reduced to 1% in the HM population while the same allele was increased to 74% in the LM population. This allele would be considered an unfavorable allele when selecting for high methionine concentration. Similarly, a *Cys2* allele that started at 8% was increased to 29% in the HM population and was not detectable in the LM population. This allele would be considered a favorable allele. The situation is less clear at the *CgS1* allele. An allele that started at 45% was increased to 93%, but the allele frequency of the LM population was nearly the same as the starting population.

## Discussion

Recurrent selection has been shown to be an effective method for altering methionine concentration in maize grain [13]. Populations resulting from these divergent selection breeding programs provide a unique opportunity for understanding how methionine concentration is controlled because they have extreme differences in grain methionine concentration while being derived from a common starting population. By comparison of high and low methionine populations at the molecular level, we sought to better understand the physiological and genetic processes controlling methionine concentration.

Mechanisms controlling grain methionine concentration can be divided into source and sink effects. We attempted to study both types of effects, including transcript levels and allele frequencies of candidate genes involved in sulfur assimilation and methionine biosynthesis as well as seed storage protein transcript and protein levels. We observed differences in both source and sink candidate genes, leading us to conclude that selection for grain methionine concentration resulted in differences for both source and sink systems. We did not characterize enough genes to draw general conclusions about relative importance of source and sink effects. Analysis of other tissues would also be helpful in assigning relative importance to source and sink effects.

The most striking difference in source genes occurred for *Cys2* and *CgS1* with changes in allele frequency, while in sink genes transcript and protein level differences were identified. Interestingly, *Cys2* was a gene identified as lacking genetic diversity in a study of 14 inbred lines selected for maximum allelic diversity [3]. In contrast, we found five different alleles in the portion of *Cys2* that we sequenced in BS11HMC6. Several of these alleles are present at low frequency. This result illustrates that collections of inbred lines may not represent the diversity present in breeding populations.

We characterized grain composition to understand the effects of selection on the methionine sink. Several changes were evident when comparing amino acid levels between the high and low methionine populations. Methionine was changed by the largest amount, with increases in the HM/LM ratio in both populations. The amino acid with the next largest change was cysteine, also a sulfur containing amino acid, however the direction of change in cysteine content was not consistent across populations. Methionine shares a common biosynthetic pathway with lysine and threonine. Interestingly, the HM/LM ratio for lysine is less than one in both the BS11- and BS31- derived populations, while the HM/LM ratio for threonine is less than one in the BS11- derived population. These reductions could be because nitrogen is limited in the seed, requiring resources to be shifted from the lysine and threonine branches of the pathway to support increased synthesis of methionine. The HM/LM ratio for nitrogen was greater than one in one selection program and less than one in the other, indicating that an increase in nitrogen is not a strict requirement for increasing methionine content. On the other hand, sulfur had HM/LM ratios greater than one in both selection programs. This is consistent with the increase in methionine content, and suggests that either sulfur uptake and/or assimilation are increased in the HM populations relative to the LM populations or that sulfur is differentially distributed in the plant between the HM and LM populations. Within the seed, the cysteine-rich 27 kDa gamma zein had lower transcript levels in the HM population in one season, and levels of this protein were lower as well. The two methionine-rich delta zein genes had the greatest increase in transcript levels comparing HM to LM and the corresponding proteins were increased as well. The 18 kDa delta zein showed the greatest increase in both transcript and protein levels. Transcript and protein levels of the 10 kDa delta zein both increased. This protein is increased in a high methionine mutant called *dzr1*[5]. Interestingly, this increase has been shown to be due to post-transcriptional regulation [9] resulting in differences in transcript stability [12]. The two alpha zeins examined at the transcript level did not change. Some alpha zein proteins increased while others decreased. Taken together, the seed protein data suggest sulfur limits methionine accumulation in the seed in spite of changes in total sulfur content, therefore sulfur is redistributed from cysteine-rich proteins into methionine-rich proteins.

We chose to focus this study on grain tissues, but it is very likely that biochemical changes in other tissues play a role in determining grain methionine concentration. Roots and leaves are known to be key organs for sulfur assimilation and would therefore be expected to influence grain methionine concentration. This may explain why we saw only minor differences in transcript levels of sulfur assimilation and methionine biosynthesis genes in grain, while the same genes showed large differences in allele distribution. It may be that the different alleles have different expression in tissues where sulfur assimilation is more active, such as roots and leaves. Selection for a trait on the basis of one tissue has been shown to influence other tissues. One example of this is that selection for long ears resulted in changes in plant height [29]. Many physiological and biochemical changes have been documented in response to selection for protein concentration as well [reviewed in [30]].

Caution should be exercised when attributing the observed differences between HM and LM populations to selection, because it is very difficult to rule out genetic drift as a possible mechanism. Changes that were observed in both the BS11- and BS31-derived experiments are consistent with the responses to selection observed, but even in these cases we cannot rigorously rule out genetic drift. This does not eliminate the value of these observations for development of further hypotheses about the role of different genes in determining grain methionine concentration. For example, selection experiments are particularly valuable for identifying rare alleles of potential importance. We observed an allele of *Cys2* that was enriched from 8% to 29% in the course of selection for HM, while it was reduced to 0% in the course of selection for LM. The low frequency of this allele in the starting population makes it unlikely that this allele would be identified as a favorable allele in a study of association between methionine content and genetic loci in the starting population, but it clearly exhibits the characteristics of an allele that was altered by selection such as this study. Thus, selection experiments provide an excellent complement to association studies for the identification of valuable rare alleles.

The use of PCA as a tool for visual representation of genetic data can contribute useful insight into selection processes over time and has been used in various forms for population structure inference [31,32]. Principal components one, two, and three separated the BS11- and BS31-derived populations, HM and LM within BS11, and HM and LM with BS31, respectively. The separation of the populations in such a way can suggest what properties of the populations account for most of the variation in the SNP data. These results suggest that differentiation of the BS11- and BS31-derived populations accounts for a majority of the variation in the data. This result can most likely be explained by the initial allelic composition of the synthetics used for population development. Results for PC2 and PC3 suggest that more of the variation in the data is explained by differentiation within BS11 than within BS31.

In agreement with our candidate regions, results for *dzs18* and *dzs10* showed a consistently strong association with methionine concentration for the BS11-derived population across cycles of selection. The lack of identified SNPs for the BS31-derived population is most likely due to the fewer number of SNPs used for that population. Another possible explanation is that the variation within BS31, separated by PC3, is considerably lower as compared within BS11. Thus, the few number of SNPs identified as significant could be explained by the lower level of variation within the BS31-derived population. There was commonality for significant SNPs within BS31 where zp27.4 was significant in two cycles of selection. This SNP is in the gene encoding the 27 kDa gamma zein. The separation of the derived populations with respect to PC1 indicates that selection within the derived populations occurs by different mechanisms. Thus, this could explain the lack of common significant SNPs between derived populations.

Linkage disequilibrium blocks between SNPs can represent regions in the genome with a high level of co-inheritance and therefore co-selective pressure. The level of LD, measured as *r*^*2*^, was calculated for the significant SNPs in the BS11-derived population and various LD blocks were observed depending on the particular cycle of selection. As expected, high LD existed between unlinked loci in all cycles of selection for the significant SNPs. In the calculation of LD between significant SNPs, two of the significant SNPs were fixed in C5, an indication of selection at those loci.

## Conclusions

Taken together, our data support a model in which selection for high grain methionine concentration increased sink strength though transcriptional up-regulation of methionine-rich zein. We cannot rule out the possibility of changes in free methionine or compounds related to S-adenosylmethionine. The additional sulfur required for this increased sink may have come from cysteine contained in the 27 kDa gamma zein gene, since the level of this protein is reduced. However, since levels of total cysteine are not reduced substantially and total sulfur levels are increased, reduction of cysteine-rich storage proteins is not a sufficient explanation. Additional sulfur in the seed is probably contributed by increased sulfur uptake and/or assimilation or from other sulfur sources within the plant. In support of this idea, a SNP near the *CgS1* locus was associated with HM/LM classification. In addition, some sulfur assimilation genes were transcriptionally up-regulated in the endosperm for one experiment and different alleles of *CgS1* and *Cys2* are present in the high and low populations. More research is needed to definitively establish the source of sulfur that accumulates in the high methionine zeins.

## Abbreviations

SNP: Single nucleotide polymorphism; HM: High methionine; LM: Low methionine; LD: Linkage disequilibrium; PC: Principal component; C#: Cycle of selection; FDR: False discovery rate; PCA: Principal component analysis.

## Competing interests

The authors declare that they have no competing interests.

## Authors’ contributions

MN carried out the association analysis. KV coordinated genotyping and analyzed genotype data. MA carried out RT-PCR. NA Sequenced alleles of candidate genes. AB analyzed alcohol soluble proteins by HPLC. MA carried out RT-PCR. AL carried out RT-PCR, methionine assays and sequencing of candidate genes. PS conceived the study and coordinated the work. All authors contributed to writing and approved the final manuscript.

## Supplementary Material

Additional file 1 Populations used in this study and genotyping strategy. Click here for file

Additional file 2 Map of SNP markers used in study. Click here for file
