# Supplementary figures and images for: Genetic and biochemical differences in populations bred for extremes in maize grain methionine concentration
Source: BMC Plant Biol. 2014 Feb 19;14:49. doi: 10.1186/1471-2229-14-49 (PMC3946590; doi:10.1186/1471-2229-14-49)

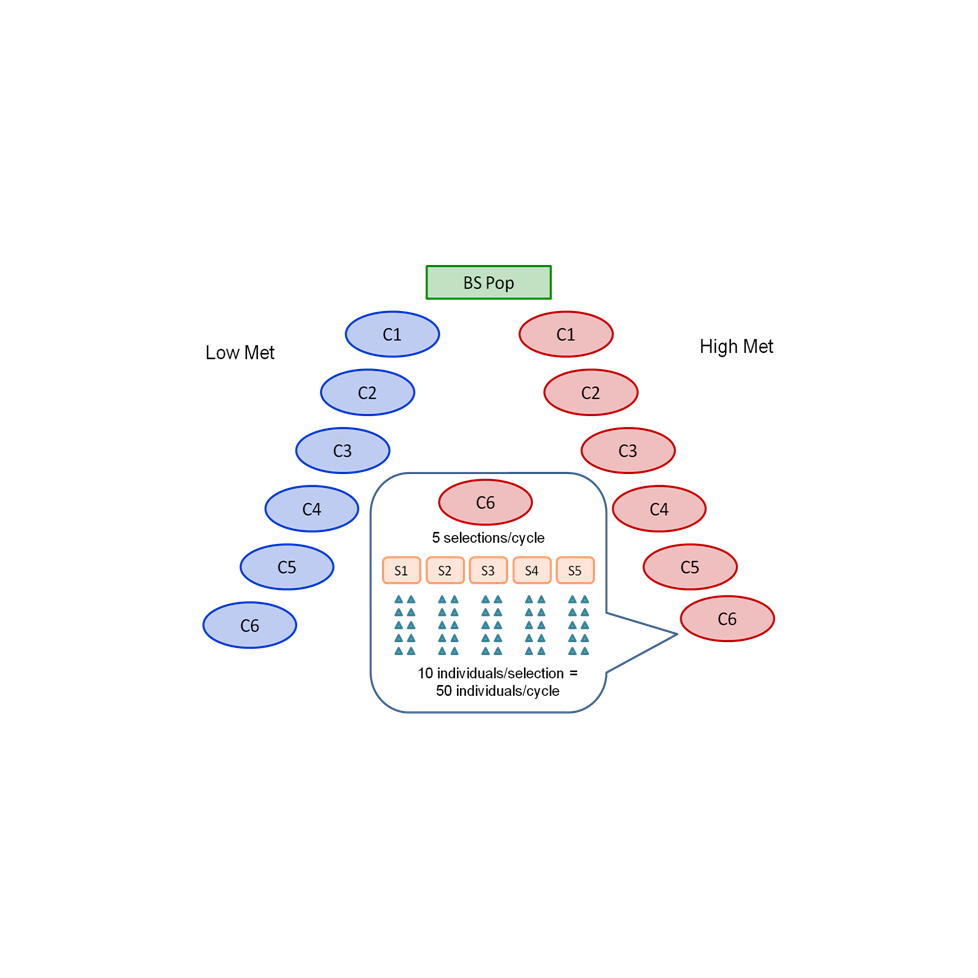

Supplement: Additional file 1 — Populations used in this study and genotyping strategy. [file 1471-2229-14-49-S1.tiff]

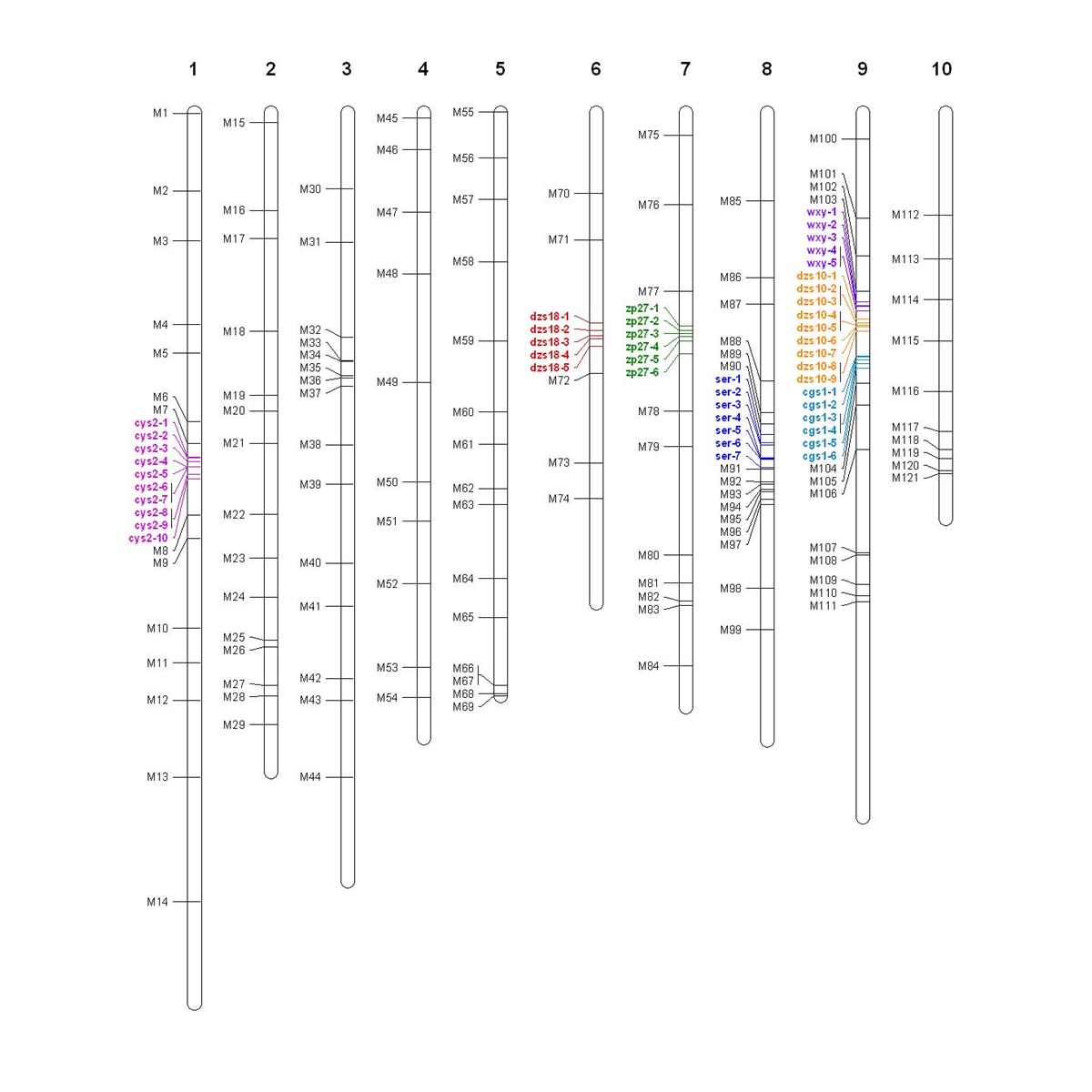

Supplement: Additional file 2 — Map of SNP markers used in study. [file 1471-2229-14-49-S2.tiff]
